# Methods for Population-Adjusted Indirect Comparisons in Health Technology Appraisal

**DOI:** 10.1177/0272989X17725740

**Published:** 2017-08-19

**Authors:** David M. Phillippo, Anthony E. Ades, Sofia Dias, Stephen Palmer, Keith R. Abrams, Nicky J. Welton

**Affiliations:** School of Social and Community Medicine, University of Bristol, Bristol, UK (DMP, AEA, SD, NJW); Centre for Health Economics, University of York, York, UK (SP); Department of Health Sciences, University of Leicester, Leicester, UK (KPA)

**Keywords:** comparative effectiveness, indirect comparison, individual patient data, population adjustment

## Abstract

Standard methods for indirect comparisons and network meta-analysis are based on aggregate data, with the key assumption that there is no difference between the trials in the distribution of effect-modifying variables. Methods which relax this assumption are becoming increasingly common for submissions to reimbursement agencies, such as the National Institute for Health and Care Excellence (NICE). These methods use individual patient data from a subset of trials to form population-adjusted indirect comparisons between treatments, in a specific target population. Recently proposed population adjustment methods include the Matching-Adjusted Indirect Comparison (MAIC) and the Simulated Treatment Comparison (STC). Despite increasing popularity, MAIC and STC remain largely untested. Furthermore, there is a lack of clarity about exactly how and when they should be applied in practice, and even whether the results are relevant to the decision problem. There is therefore a real and present risk that the assumptions being made in one submission to a reimbursement agency are fundamentally different to—or even incompatible with—the assumptions being made in another for the same indication. We describe the assumptions required for population-adjusted indirect comparisons, and demonstrate how these may be used to generate comparisons in any given target population. We distinguish between anchored and unanchored comparisons according to whether a common comparator arm is used or not. Unanchored comparisons make much stronger assumptions, which are widely regarded as infeasible. We provide recommendations on how and when population adjustment methods should be used, and the supporting analyses that are required to provide statistically valid, clinically meaningful, transparent and consistent results for the purposes of health technology appraisal. Simulation studies are needed to examine the properties of population adjustment methods and their robustness to breakdown of assumptions.

Standard methods for indirect comparisons^1^ and network meta-analysis^2^ (NMA) (see Dias et al.^3^ for a comprehensive guide) are based on aggregate data, and assume that the distributions of effect-modifying variables do not differ between studies. Methods that relax this assumption to form “population-adjusted indirect comparisons” are becoming increasingly common for submissions to reimbursement agencies, such as the National Institute for Health and Care Excellence (NICE). Ideally, we would have individual patient data (IPD) from all studies to fully adjust for patient differences using network meta-regression, as aggregate data network meta-regression has low power to detect or adjust for covariates and is susceptible to ecological bias.^4,5^ However, it is rarely the case that full IPD are available. In particular, a very common scenario is when a company has IPD on its own trial but only published aggregate data on their competitor’s trial, typically consisting of average treatment effects and summary patient characteristics (e.g., mean and standard deviation for continuous characteristics, and proportions for binary/categorical). Population adjustment methods use the available IPD to adjust for between-trial imbalances in the distribution of observed covariates. These methods cannot adjust for differences in, for example, treatment administration, co-treatments, or treatment switching, as these are perfectly confounded with treatment. We focus on 2 recently proposed methods: Matching-Adjusted Indirect Comparison (MAIC)^6–8^ and Simulated Treatment Comparison (STC).^7,9^ MAIC and STC are not the only possible approaches to population adjustment; we outline some alternatives in the discussion.

This paper is based on a Technical Support Document prepared for the NICE Decision Support Unit, available from http://www.nicedsu.org.uk/.^10^ We begin by introducing the population adjustment scenario. We then describe MAIC and STC in detail, and clearly set out their assumptions and properties. We propose the shared effect modifier assumption which, if justified, may be used to transport indirect comparisons into any target population. Recommendations on the use of population adjustment methods in technology appraisal are then given, with a particular focus on reproducibility, consistency, and transparency, whilst minimizing bias and maximizing precision.

## Overview of the Problem

We focus exposition on a simple indirect comparison between 2 treatments based on 2 studies; although, our recommendations and many of the methods are generalizable to comparisons involving more treatments or studies.^10^ We distinguish between population adjustment methods to make “anchored” indirect comparisons, where the evidence is connected by a common comparator, and “unanchored” indirect comparisons, where the evidence is disconnected due to a lack of a common comparator or single-arm studies. We begin by describing the anchored scenario; the unanchored scenario then follows simply (albeit, with very different assumptions; see the overview in the following section). We make a clear and necessary distinction between prognostic variables and effect modifiers: prognostic variables are covariates that affect the outcome whereas effect modifiers (also known as predictive variables^11^) are covariates that alter the effect of treatment as measured on a given scale. Effect modifiers are not necessarily also prognostic variables, and may be specific to each treatment. Effect modifier status on one scale does not necessarily imply effect modifier status on another scale. We assume internal validity of the studies included in the analysis, so that the studies provide unbiased estimates of treatment effects in their respective sample populations.

Consider one AB trial, for which the analyst has IPD, and one AC trial, for which only published aggregate data are available. We wish to estimate a comparison of the effects of treatments B and C on an appropriate scale in some target population P, denoted by the parameter $d_{BC(P)}$. Within the AB trial population, there are parameters $\mu_{A(AB)}$, $\mu_{B(AB)}$ and $\mu_{C(AB)}$ representing the expected outcome on each treatment (including for treatment C, which was not studied in the AB trial). The AB trial provides estimators ${\bar{Y}}_{A(AB)}$ and ${\bar{Y}}_{B(AB)}$ of $\mu_{A(AB)}$ and $\mu_{B(AB)}$, respectively, which are the summary outcomes; for example, the probability of success, on each arm (note that $\mu_{C(AB)}$ is not estimated by the AB trial). There is a parallel system of parameters ($\mu_{A(AC)},\;\mu_{B(AC)},\;\mu_{C(AC)}$) and estimators (${\bar{Y}}_{A(AC)},\;{\bar{Y}}_{C(AC)}$) in the AC trial.

Having selected a suitable scale, for example, a logit, log, risk difference, or mean difference scale, we form estimators ${\hat{\Delta }}_{AB(AB)}$ and ${\hat{\Delta }}_{AC(AC)}$ of the population-specific relative treatment effects $d_{AB(AB)}$ and $d_{AC(AC)}$ in each trial using the appropriate link function g(·):

$${\hat{\Delta }}_{AB(AB)}=g\left({\bar{Y}}_{B(AB)}\right)-g\left({\bar{Y}}_{A(AB)}\right),\;{\hat{\Delta }}_{AC(AC)}=g\left({\bar{Y}}_{C(AC)}\right)-g\left({\bar{Y}}_{A(AC)}\right)$$

Standard methods for indirect comparisons make the assumption that there is no difference in the distribution of trial-level effect modifiers, specific to the chosen scale, between the populations in the AB and AC trials or the target population P, so that population-specific relative treatment effects are equal across populations: $d_{AB(AB)}=d_{AB(AC)}=d_{AB(P)}$ and $d_{AC(AB)}=d_{AC(AC)}=d_{AC(P)}$. Under this assumption, the standard indirect comparison estimator of the relative effect $d_{BC(P)}$ is

$${\hat{\Delta }}_{BC(P)}={\hat{\Delta }}_{AC(AC)}-{\hat{\Delta }}_{AB(AB)},$$

which takes account of the fact that patients are only randomized within trials.^1^

The final step is to apply these relative effects to a specified target population P in which the summary absolute effect (such as the mean change from baseline, or probability of response) of treatment A is ${\bar{Y}}_{A(P)}$. We can now estimate the summary absolute effects of treatments A,B,C in the target population, $\mu_{A(P)},\;\mu_{B(P)},\;\mu_{C(P)}$, which have estimators

$${\bar{Y}}_{A(P)},\;{\hat{Y}}_{B(P)}=g^{-1}\left(g\left({\bar{Y}}_{A(P)}\right)+{\hat{\Delta }}_{AB(P)}\right),{\hat{Y}}_{C(P)}=g^{-1}\left(g\left({\bar{Y}}_{A(P)}\right)+{\hat{\Delta }}_{AC(P)}\right).$$

Suppose that in each trial we have information on a common set of covariates X. Between-trial differences in the distribution of prognostic variables that are not effect modifiers do not affect inference, because the within-trial randomization means that they do not impact on the relative treatment effects (assuming that the sample size is sufficiently large). Note that effect modifiers $X^{EM}$, a subset of X, are assumed to have an additive effect on the transformed scale, such that, at any given value of $X^{EM}$, the conditional relative effect is $d_{AB}\left(X^{EM}\right)=d_{AB}\left(0\right)+\gamma^{T}X^{EM}$, conceptualized as an “intercept” term (the relative effect $d_{AB}\left(0\right)$ at $X^{EM}=0$) plus an interaction effect, $\gamma^{T}X^{EM}$.

If there are effect modifiers and if these are distributed differently between the populations, the relative treatment effects $d_{AB(AB)},d_{AC(AC)}$ that can be estimated directly from each trial are only valid for a population with the distribution of effect modifiers observed in that trial. For example, we would have estimates ${\hat{d}}_{AB(AB)}$ in the AB population and ${\hat{d}}_{AC(AC)}$ in the AC population, but it would not be possible to identify a coherent set of estimates, either for the population represented in the AB trial

$${\hat{d}}_{AB(AB)},\;{\hat{d}}_{AC(AB)},\;{\hat{d}}_{BC(AB)}={\hat{d}}_{AC(AB)}-{\hat{d}}_{AB(AB)},$$

or for the population represented in the AC trial

$${\hat{d}}_{AB(AC)},\;{\hat{d}}_{AC(AC)},\;{\hat{d}}_{BC(AC)}={\hat{d}}_{AC(AC)}-{\hat{d}}_{AB(AC)},$$

or, indeed, for any other target population.

The premise of MAIC and STC is to “adjust for” between-trial differences in “baseline characteristics”, in order to identify a coherent set of estimates where standard methods of indirect comparison cannot. Both methods use IPD on the AB trial to form predictors ${\hat{Y}}_{A(AC)},\;{\hat{Y}}_{B(AC)}$ of the summary outcomes that would be observed on treatments A and B in the AC trial if the AB trial population was the same as the AC trial population.

The predicted outcomes ${\hat{Y}}_{A(AC)},{\hat{Y}}_{B(AC)}$ may then be used in 2 ways. First, relative effects may be estimated by an anchored indirect comparison:

$${\hat{\Delta }}_{BC(AC)}=\left(g\left({\bar{Y}}_{C(AC)}\right)-g\left({\bar{Y}}_{A(AC)}\right)\right)-\left(g\left({\hat{Y}}_{B(AC)}\right)-g\left({\hat{Y}}_{A(AC)}\right)\right).$$

Alternatively, an unanchored indirect comparison can generated:^7,8^

$${\hat{\Delta }}_{BC(AC)}=g\left({\bar{Y}}_{C(AC)}\right)-g\left({\hat{Y}}_{B(AC)}\right).$$

The anchored indirect comparison should always be preferred in a connected network as it respects the randomization within studies, whereas the unanchored indirect comparison requires much stronger assumptions that are very hard to meet. If the treatment network is disconnected or contains single-arm studies, then there is no common comparator arm through which to make an anchored indirect comparison, and we are obliged to rely on an unanchored indirect comparison.

MAIC and STC are both based upon methods that date back several decades—propensity score reweighting and regression adjustment, respectively—and are discussed extensively in the literature on standardization,^12–15^ generalization,^16–20^ and calibration.^21–24^ Like MAIC and STC, these methods have been aimed at mapping the absolute and relative effects observed in one population into effects that would be predicted in another, in both randomized and observational study settings. The novel aspect of MAIC and STC is to provide indirect comparisons when IPD are only available in the AB trial, with aggregate data in the AC trial along with summary information on the covariate distribution. If individual patient data are available on both the AB and AC studies, a network meta-regression using IPD is the gold-standard approach.^4;5;25-27^ Ideally, the full joint distribution of covariates X is known or can be obtained from conditional distributions, but frequently in practice only the marginal mean and standard deviation of each covariate are known. Due to the lack of IPD from the AC trial, standard approaches to fitting both propensity score and outcome models cannot be used. We outline both MAIC and STC approaches below. A worked example of MAIC and STC as conforming to our recommendations is provided in the online Appendix.

## Overview of Methods for Population Adjustment with Limited IPD

### Population Reweighting Methods

MAIC is a reweighting method similar to inverse propensity score weighting^14^ and non-parametric likelihood reweighting,^23^ which allows the propensity score logistic regression model to be estimated without IPD in the AC population. The mean outcomes $\mu_{t(AC)}$ on treatment t=A,B in the AC target population are estimated by taking a weighted average of the outcomes $Y_{it(AB)}$ of the $N_{t(AB)}$ individuals in arm t of the AB population,

$${\hat{Y}}_{t(AC)}=\frac{\overset{N_{t(AB)}}{\underset{i=1}{\sum }}Y_{it(AB)}w_{it}}{\overset{N_{t(AB)}}{\underset{i=1}{\sum }}w_{it}},$$

where the weight $w_{it}$ assigned to the i-th individual receiving treatment t is equal to the odds of being enrolled in the AC trial v. the AB trial. The weights are estimated using logistic regression as $\log\left(w_{it}\right)=\alpha_{0}+\alpha_{1}^{T}X_{it}$, where $X_{it}$ is the covariate vector for the i-th individual receiving treatment t; however, the regression parameters are not estimable using standard methods due to the lack of IPD in the AC trial. Signorovitch et al.^6^ use the method of moments to estimate ${\hat{\alpha }}_{1}$ so that the weights exactly balance the mean covariate values (and any included higher order terms; for example, squared covariate values to balance the variance) between the weighted AB population and the AC population. When ${\bar{X}}_{\left(AC\right)}=0$, Signorovitch et al. show that this is equivalent to minimizing $\sum_{t=A,B}\sum_{i=1}^{N_{t(AB)}}\exp\left(\alpha_{1}^{T}X_{it}\right)$. The estimator in equation (6) is then equal to

$${\hat{Y}}_{t(AC)}=\frac{\overset{N_{t(AB)}}{\underset{i=1}{\sum }}Y_{it(AB)}\exp\left({\hat{\alpha }}_{1}^{T}X_{it}\right)}{\overset{N_{t(AB)}}{\underset{i=1}{\sum }}\exp\left({\hat{\alpha }}_{1}^{T}X_{it}\right)},$$

noting that $\exp\left({\hat{\alpha }}_{0}\right)$ cancels from the top and bottom of the fraction. Anchored and unanchored indirect comparisons are then formed using equations (4) and (5), respectively. Although MAIC can be used to facilitate indirect comparisons on any scale, the MAIC literature almost exclusively performs comparisons on the natural outcome scale (i.e., with g(·) the identity function). Typically, standard errors for MAIC estimates are calculated using a robust sandwich estimator^28^ (see the Appendix of Signorovitch et al.^6^). Sandwich estimators are derived empirically from the data, and account for the fact that the weights are estimated rather than fixed and known. Signorovitch et al.^6^ suggest reporting the effective sample size (ESS) of the pseudo-population formed by weighting the AB population, approximated by

$$\mathrm{ESS}={\left(\sum_{t=A,B}\sum_{i=1}^{N_{t(AB)}}{\hat{w}}_{it}\right)}^{2}/\sum_{t=A,B}\sum_{i=1}^{N_{t(AB)}}{\hat{w}}_{it}^{2}.$$

This approximate ESS is only accurate if the weights are fixed and known, or if they are uncorrelated with the outcome—neither of which is true here. As such, this approximation is likely to be an underestimation of the true ESS.^29^ Small ESS indicates that the weights are highly variable due to a lack of population overlap, and that the resulting estimate may be unstable. The distribution of weights themselves should also be examined directly, to diagnose issues with a lack of population overlap and to highlight any overly influential individuals. It is not possible to apply traditional propensity score tools for “balance checking” here,^19,20^ as propensity scores are only estimated for the AB trial, and the method of moments, by definition, ensures covariate balance (at least to the level of information published in the AC trial).

Another form of population reweighting is based on entropy balancing,^30^ and was first suggested for treatment effect calibration by Belger et al.^31,32^ The approach is identical to standard MAIC except that the weights are additionally constrained to be as close to each other as possible; entropy balancing methods should thus have equal or reduced standard error compared to MAIC, whilst achieving the same reduction in bias. Different schemes for applying weights have also been proposed.^31,32^ These involve splitting apart trial arms and balancing covariate distributions separately between the control arms (A) and between the treatment arms (B and C) in the IPD and aggregate populations. The properties of such “splitting” approaches in comparison with more typical population reweighting are largely unknown, and require further investigation.

### Outcome Regression Methods

Simulated Treatment Comparison (STC) is a modification of covariate adjustment,^21^ which fits an outcome model using the IPD in the AB trial:

$$g\left(\mu_{t(AB)}\left(X\right)\right)=\beta_{0}+\beta_{1}^{T}X+\left(\beta_{B}+\beta_{2}^{T}X^{EM}\right)I\left(t=B\right),$$

where $\beta_{0}$ is an intercept term, $\beta_{1}$ is a vector of coefficients for prognostic variables, $\beta_{B}$ is the relative effect of treatment B compared to A at X=0, $\beta_{2}$ is a vector of coefficients for effect modifiers $X^{EM}$ (a subvector of the full covariate vector X), and $\mu_{t(AB)}\left(X\right)$ is the expected outcome of an individual assigned treatment t with covariate values X, which is transformed onto a chosen linear predictor scale with link function g(·).

The model in equation (8) is a more general form of that given by Ishak et al.^7^, which does not include any effect modifier terms. The STC literature advocates forming indirect comparisons directly on the natural outcome scale with g(·) the identity link in equation (4) or (5); however, this leads to scale conflicts^10^ if the same link is not used in the outcome model (8) (see the following section on the importance of scale). ${\hat{Y}}_{A(AC)}$ and ${\hat{Y}}_{B(AC)}$ may be predicted from the outcome regression by substituting in mean covariate values to obtain ${\hat{Y}}_{A(AC)}=g^{-1}({\hat{\beta }}_{0}+{\hat{\beta }}_{1}^{T}{\bar{X}}_{\left(AC\right)})$ and ${\hat{Y}}_{B(AC)}=g^{-1}({\hat{\beta }}_{0}+{\hat{\beta }}_{1}^{T}{\bar{X}}_{\left(AC\right)}+{\hat{\beta }}_{B}+{\hat{\beta }}_{2}^{T}{\bar{X}}_{\left(AC\right)}^{EM})$. These estimators are systematically biased whenever g(·) is not the identity function, because the mean outcome depends on the full distribution of the covariates and not just their mean.^7^ Instead of substituting in mean covariate values in this case, Ishak et al. suggest that estimates are obtained by first drawing samples from the joint covariate distribution in the AC trial and then averaging over the predicted outcomes based on the regression model. This simulation approach, however, inflates uncertainty of the relative effect estimates.

Standard tools for model checking (such as AIC/DIC, examining residuals, among others) may be used when constructing the outcome model in the AB trial; however (as with MAIC), additional assumptions are required to predict absolute outcomes in the AC population, which are difficult to test with the limited data available.

### Overview of Assumptions Made by Different Methods

It is critical to note that unanchored indirect comparisons require much stronger assumptions than anchored indirect comparisons. The assumptions required by different forms of population-adjusted indirect comparisons are summarized in Table 1.

**Table 1:** Assumptions Made by Different Methods for Indirect Comparisons

	Method					
Assumptions Made	Standard Indirect Comparison, NMA	Network Meta-regression^a^	Unanchored MAIC	Anchored MAIC	Unanchored STC	Anchored STC
**Constancy**						
**Constancy of absolute effects**	N	N	N	N	N	N
**Conditional constancy of absolute effects**	N	N	Y Typically on natural outcome scale.	N	Y Typically on natural outcome scale.	N
**Constancy of relative effects**	Y On linear predictor scale. For RE NMA relaxed to constancy in expectation.	N	N	N	N	N
**Conditional constancy of relative effects**	N	Y On linear predictor scale.	N	Y Typically on natural outcome scale.	N	Y Typically on natural outcome scale.
**Shared effect modifiers**	N/A	Y On linear predictor scale. Not required if IPD are available on both studies.	N^b^	N^b^	N^b^	N^b^

a The assumptions set out here are applicable to all forms of network meta-regression with varying combinations of IPD and aggregate data (both studies IPD, both studies aggregate data, one IPD and one aggregate), with the exception of the shared effect modifier assumption which is not required if IPD are available on both studies.

b The shared effect modifier assumption is not required, but may be additionally assumed in order to present estimates for another target population.

A standard indirect comparison or (fixed effect) network meta-analysis assumes “constancy of relative effects” on the linear predictor scale, meaning that the expected relative C v. A effect in the AC trial is identical to that which would be expected in the AB trial. This requires that any and all effect modifiers are balanced between the 2 trial populations.

Anchored forms of population-adjusted indirect comparisons rely on “conditional constancy of relative effects,” typically on the natural outcome scale. This means that the relative treatment effects are assumed constant between studies at any given level of the effect modifiers, so there is no imbalance of unobserved effect modifiers between the 2 trial populations. This is quite a strong assumption but considerably less strong than the constancy of relative effects assumption required for a standard indirect comparison.

Unanchored forms of population-adjusted indirect comparisons make the much stronger assumption of “conditional constancy of absolute effects.” This means that the absolute treatment effects are assumed constant at any given level of the effect modifiers and prognostic variables, and all effect modifiers and prognostic variables are required to be known. This is a far more demanding assumption than either constancy or conditional constancy of relative effects, and widely accepted to be very hard to meet.

The assumptions of internal validity and some form of constancy are sufficient in the scenario where, despite not having access to IPD on the AC trial, sufficient information on the joint covariate distribution is available. In practice, even this level of detail is unlikely, as published trials frequently report only details of the marginal covariate distributions (e.g., mean/median and standard deviation for continuous covariates, or proportion of individuals with a binary/categorical trait). Additional assumptions are therefore required: either that the true outcome model does not depend on the correlations between covariates, or that the missing correlations in the AC trial may be imputed from those observed in the AB trial.^10^

### The Importance of Scale and its Relation to Effect Modification

The standard practice for indirect comparisons, in common with standard methods of meta-analysis, is that they are made on a pre-specified transformed scale (e.g., on the log scale for odds ratios and risk ratios), rather than on the natural outcome scale;^1,3^ to aid interpretation or for the purposes of a cost effectiveness analysis, the resulting estimates are back-transformed onto the natural scale. The reasons for this choice include approximate normality and the stabilization of variance. Critically, for indirect comparisons, effects are assumed to be additive and linear on the transformed scale.

Effect modifier status is scale-specific,^33^ and the status of a variable as an effect modifier on one scale does not imply (either positively or negatively) the effect modifier status on any other scale. MAIC and STC, as currently practiced, are typically carried out on the natural outcome scale, regardless of the conventional linear predictor scale, so that variables that are effect modifiers in a standard indirect comparison might not be in MAIC/STC, and vice versa. Furthermore, forming the indirect comparison on a different scale to that used for the outcome model in STC results in serious issues due to the conflicting scales: linearity and additivity cannot hold on both scales, the definition of effect modifiers is obscured, and the subsequent indirect comparison is uninterpretable. The choice of an appropriate scale is therefore critical, and should be made using biological and clinical knowledge.^34^ Moreover, where a standard scale exists for a given outcome upon which additivity is commonly accepted, the use of an alternative scale is hard to justify.

In a decision-making context, the possibility of effect modification has to be handled carefully, not least because a treatment that is cost-effective at one value of the effect modifier might not be at another. Guidelines on methods recommend that effect modifiers must be pre-specified and clinically plausible, and that supporting evidence must be provided from a thorough review of the subject area or from expert clinical opinion (see Section 5.2.7 of the NICE Methods Guide,^35^ and ISPOR guidance^36^).

## Calibrating Population-Adjusted Estimates to the Correct Target Population

The premise of both MAIC and STC is that the treatment effect depends on the population. It is therefore not sufficient to use MAIC or STC to generate an “unbiased” comparison in just any population; they are only useful for decision making if they can produce a fair comparison in the target population for the decision. In general, the target population should be a cohort or registry study population relevant to the clinical decision, which is unlikely to match the population of the AC trial. However, MAIC and STC, as currently proposed, are unable to achieve estimates in any population other than that of the AC study.

To allow relative treatment effects to be projected into any target population, we propose that an additional assumption is made, known as the “shared effect modifier assumption.” The shared effect modifier assumption applies to a set of active treatments T, and states that 1) the effect modifiers of all treatments in T are the same, and 2) the change in treatment effect caused by each effect modifier is the same for all treatments in T. This assumption is not required for MAIC or STC as currently used. However, if deemed reasonable, it may be leveraged to produce indirect comparisons in any given target population. For example, if the shared effect modifier assumption holds for treatments B and C, then the estimated $d_{BC}$ relative treatment effect (whether obtained using anchored or unanchored MAIC/STC) will be applicable to any population. In general, we make use of the relationship

$$d_{tu(P)}=d_{tu(Q)}\;\forall t,u\in T$$

for any 2 populations P and Q and for a set of treatments T for which the shared effect modifier assumption holds.

Mathematical proof and examples are provided in Appendix A. The shared effect modifier assumption is evaluated on a clinical and biological basis; treatments in the same class (i.e., sharing biological properties or mode of action) are more likely to satisfy the shared effect modifier assumption than those from different classes.

## Recommendations for the use of Population-Adjusted Indirect Comparisons

The exact properties of population adjustment methodologies, such as MAIC and STC, in anchored and unanchored forms and their performance relative to standard indirect comparisons can only be properly assessed by a comprehensive simulation exercise. For this reason, we do not express preference for any particular population adjustment method. However, based on general principles, we can draw some useful conclusions about the role of population-adjusted estimates of treatment effects, including the types proposed by MAIC and STC, in submissions to reimbursement agencies.

The recommendations in Table 2 and reporting guidelines in Appendix B are intended to promote reproducibility, consistency, and transparency in the use of population adjustment methods, whilst minimizing bias and maximizing precision. A further desirable property is that, if there were no effect modifiers, no adjustment would occur: the estimates would be expected to be exactly those produced by standard indirect comparison. Appendix C provides flow charts summarizing these recommendations, and describing the process of selecting a method for indirect comparison, undertaking the analysis, and presenting the results.

**Table 2:** Recommendations for the Use of Population-Adjusted Indirect Comparisons

**Recommendation 1:** When connected evidence with a common comparator is available, a population-adjusted anchored indirect comparison may be considered. Unanchored indirect comparisons may only be considered in the absence of a connected network of randomized evidence, or where there are single-arm studies involved.
**Recommendation 2:** Submissions using population-adjusted analyses in a connected network need to provide evidence that they are likely to produce less biased estimates of treatment differences than could be achieved through standard methods.
(a) Evidence must be presented that there are grounds for considering one or more variables as effect modifiers on the appropriate transformed scale. This can be empirical evidence or an argument based on biological plausibility.
(b) Quantitative evidence must be presented that population adjustment would have a material impact on relative effect estimates due to the removal of substantial bias.
**Recommendation 3:** Submissions using population-adjusted analyses in an unconnected network need to provide evidence that absolute outcomes can be predicted with sufficient accuracy in relation to the relative treatment effects, and present an estimate of the likely range of residual systematic error in the “adjusted” unanchored comparison.
**Recommendation 4:** The following variables should be adjusted for in a population-adjusted analysis:
(a) For an anchored indirect comparison, propensity score weighting methods should adjust for all effect modifiers (in imbalance or not) but no prognostic variables. Outcome regression methods should adjust for all effect modifiers in imbalance, and any other prognostic variables and effect modifiers that improve model fit.
(b) For an unanchored indirect comparison, both propensity score weighting and outcome regression methods should adjust for all effect modifiers and prognostic variables to reliably predict absolute outcomes.
**Recommendation 5:** Indirect comparisons should be carried out on the linear predictor scale, with the same link functions that are usually employed for those outcomes.
**Recommendation 6:** The target population for any treatment comparison must be explicitly stated, and population-adjusted estimates of the relative treatment effects must be generated for this target population.

Recommendations 1 to 3 are concerned with choosing and justifying an appropriate form of population-adjusted indirect comparisons over a standard indirect comparison. Since unanchored comparisons make much stronger assumptions than anchored comparisons, recommendation 1 is that the latter should always be preferred. For anchored comparisons, recommendation 2 requires that a priori evidence of effect modifier status is provided, along with evidence of substantial imbalance; this stems from established guidance on effect modification.^35,36^ Unanchored comparisons cannot rely on randomization and thus are problematic (see the overview of assumptions above). Recommendation 3 for unanchored comparisons therefore calls for evidence of predictive accuracy for absolute outcomes, and an estimate of residual bias due to unaccounted for covariates, without which the amount of bias is unknown but is likely to be substantial, and could even exceed the magnitude of treatment effects being estimated.

Recommendation 4 ensures that bias is minimized whilst controlling standard error. For anchored indirect comparisons performed via population reweighting methods (e.g., MAIC), all effect modifiers should be adjusted for, whether in imbalance or not, to ensure balance and reduce bias. To avoid loss of precision due to over-matching, purely prognostic variables should not be adjusted for, as they do not affect the estimated relative treatment effect. For anchored indirect comparisons performed via outcome regression methods (e.g., STC), all effect modifiers in imbalance should be adjusted for to reduce bias. The inclusion of additional prognostic variables and effect modifiers in the model can result in a gain in precision of the estimated treatment effect if the variable accounts for a substantial degree of variation in the outcome, but will not reduce bias any further. For an unanchored indirect comparison, reliable predictions of absolute outcomes are required; therefore, population adjustment methods should adjust for all effect modifiers and prognostic variables.

Recommendation 5 is to choose an appropriate linear predictor scale for the adjustment and subsequent indirect comparison in line with general modelling practice,^35,37^ avoiding scale conflicts (see the above section on the importance of scale). If a scale is chosen that differs from what is usually used in existing literature for that outcome and condition, thorough justification must be given.

As noted in the previous section, population adjustment methods are only useful for decision making if they can produce estimates for the appropriate target population; recommendation 6 makes this explicit, and the shared effect modifier assumption defined above may be utilized if appropriate.

Further detail on each of the recommendations may be found in NICE DSU Technical Support Document 18.^10^

## Discussion

The rationale for employing population adjustment stems principally from 2 scenarios: 1) connected, comparative evidence is available but standard synthesis methods are deemed inappropriate due to an imbalance in suspected effect modifiers; or 2) no connected evidence is available, or comparisons are required involving single-arm studies. In this paper, we focused on a simple 2-study indirect comparison; however, in principle, the methods and recommendations are generalizable to situations where multiple studies are available for each comparison or involving larger treatment networks. This is a clear area for further research.

As with standard methods for indirect comparison^1^ and network meta-analysis,^3^ population-adjusted indirect comparisons assume internal validity of the included studies. An appropriately well-designed randomized study is expected to balance the distributions of both observed and unobserved prognostic variables and effect modifiers between arms. Further research is necessary to investigate methods for adjusting for within-study covariate imbalance, and other issues with internal validity, such as lack of blinding.

By definition, the presence of effect modification means that relative treatment effects may differ between populations and, as a result, different decisions could be reached in different populations. MAIC and STC apply propensity score reweighting and outcome regression to produce an indirect comparison in the aggregate data population, typically that of a competitor’s study, which is unlikely to match the decision target population. If the competitor was to use its IPD and run the analysis the other way around, apparently contradictory results could be obtained. This has already arisen in practice, with 2 MAIC analyses from competing manufacturers comparing treatments for ankylosing spondylitis.^38,39^ Each manufacturer had IPD available on their own study and used MAIC to form a comparison in their competitor’s study population, and each obtained opposing results in favor of their own treatment. With a decision target population in mind, however, we note that the real conflict lies not in the different results produced by the 2 MAICs but in deciding which of the 2 study populations better represents the decision target population. Ironically, each company is left in the position of implicitly assuming that their competitor’s trial is more representative of the decision target population than their own. We have shown that the shared effect modifier assumption, if justified, may be used to transport indirect comparisons into the target population for the decision without pleading to representativeness. Methods that relax the shared effect modifier assumption, or attempt to validate it, are areas for further research.

Much of the literature on unanchored MAIC and STC acknowledges the possibility of residual bias due to unobserved prognostic variables and effect modifiers;^40–44^ however, it is not made clear that the accuracy of the resulting estimates is entirely unknown, because there is no analysis of the potential magnitude of residual bias, and hence no idea of the degree of error in the unanchored estimates. It is, of course, most unlikely that systematic error has been eliminated. Hoaglin,^45,46^ in a critique of an unanchored comparison^47^ based upon a matching approach similar to MAIC, remarked that, without providing evidence that the adjustment compensates for the missing common comparator arms and the resulting systematic error, the ensuing results “are not worthy of consideration.” If unanchored forms of population adjustment are to be presented, it is essential that submissions to reimbursement agencies include information on the likely bias resulting from unobserved prognostic factors and effect modifiers distributed differently in the trials. The way in which residual systematic error is quantified is an area that requires further research.

A potential and oft-cited advantage of MAIC is that it is perceived to be “scale-free”, as the definition of the weighting model does not require any fixed outcome scale to be chosen.^6,7^ Although it is true that the reweighting procedure makes no scale assumptions, the subsequent indirect comparison does assume additivity on a specific scale, and therefore neither MAIC nor STC are “scale-free” in this important sense.

Setting aside their failure to generate coherent population-adjusted estimates for the chosen target population, MAIC and STC also give very considerable leeway to investigators to choose anchored or unanchored approaches, and to pick and choose variables for adjusting. In the interests of transparency and consistency, and to ensure equity for patients and a degree of certainty for those making submissions to reimbursement agencies, it is essential to regularize how and under what circumstances these procedures should be used, and which additional analyses should be presented to support their use and assist interpretation. We believe that the recommendations set out here go a long way toward meeting these objectives.

MAIC and STC are not the only approaches to population adjustment. One alternative stems from network meta-regression, with regression models defined at both the individual level and at the study level.^48–53^ If the study level model is an integration of the individual level model over the study population,^50,52,53^ then aggregation bias is avoided;^4,10,54^ however, at present, these types of models have only been derived for simple scenarios with binary covariates.^10,50^ Attractively, these methods are naturally generalizable to connected networks of any size, and they reduce to the gold standard IPD network meta-regression if IPD are available for all trials. Doubly robust techniques that combine both reweighing and regression adjustment are also plausible, and have been described for the case when full IPD are available by Zhang et al.^24^ We would expect these alternatives to have similar properties to MAIC and STC in both anchored and unanchored scenarios, and the recommendations made in the last section are applicable to population adjustment methods in general; for a more detailed discussion, see NICE DSU Technical Support Document 18.^10^ Further research is needed to assess all available methods alongside MAIC and STC; in particular, to examine their properties and robustness to breakdown of assumptions, with varying levels of data availability, through thorough simulation studies.

## Supplementary Material

Supplementary material

Supplementary material

Supplementary material
