# Supplementary material for: Methods for Population-Adjusted Indirect Comparisons in Health Technology Appraisal
Source: Med Decis Making. 2017 Aug 19;38(2):200–11. doi: 10.1177/0272989X17725740 (PMC5774635; doi:10.1177/0272989X17725740)
Supplement: Supplementary material [file AppendixA.pdf]

Appendix A: TRANSPOSING INDIRECT COMPARISONS TO OTHER TARGET POPULATIONS

Under the assumption of shared effect modifiers for a set of treatments $\mathcal{T}$, we have the relation on the population-specific relative treatment effects from equation (9):

$$d_{tu(P)} = d_{tu(Q)} \quad \forall t,u \in \mathcal{T}$$

which holds for any two populations P and Q.

Proof

Using additivity on an appropriate linear predictor scale, we write the transformed conditional absolute treatment effects $\eta_t\left(\boldsymbol{X},\boldsymbol{U}\right)$ as

$$\eta_t\left(\boldsymbol{X},\boldsymbol{U}\right) = \beta_0 + \boldsymbol{\beta}_1^T\boldsymbol{X} + \boldsymbol{\varphi}_1^T\boldsymbol{U} + \left(\beta_t + \boldsymbol{\beta}_{2,t}^T\boldsymbol{X}^{EM} + \boldsymbol{\varphi}_{2,t}^T\boldsymbol{U}^{EM}\right)I\left(t \neq A\right), \tag{10}$$

where $\boldsymbol{X}$ and $\boldsymbol{U}$ are vectors of observed and unobserved covariates respectively (possibly with interactions or higher order terms), with corresponding subvectors of effect modifiers $\boldsymbol{X}^{EM}$ and $\boldsymbol{U}^{EM}$. Equation (10) represents the underlying (transformed) outcome model, which cannot be estimated directly as $\boldsymbol{U}$ are unobserved.

Using the shared effect modifier assumption on the set of treatments $\mathcal{T}$, which means that $\boldsymbol{\beta}_{2,t} = \boldsymbol{\beta}_2$ and $\boldsymbol{\varphi}_{2,t} = \boldsymbol{\varphi}_2$ $\forall t \in \mathcal{T}$ we rewrite the outcome model (10) for $t \in \mathcal{T}$ as

$$\eta_t\left(\boldsymbol{X},\boldsymbol{U}\right) = \beta_0 + \boldsymbol{\beta}_1^T\boldsymbol{X} + \boldsymbol{\varphi}_1^T\boldsymbol{U} + \left(\beta_t + \boldsymbol{\beta}_2^T\boldsymbol{X}^{EM} + \boldsymbol{\varphi}_2^T\boldsymbol{U}^{EM}\right)I\left(t \neq A\right). \tag{11}$$

We then write the relative effects between any two treatments $t,u \in \mathcal{T}$ in any two populations P and Q using equation (11) as

$$\begin{aligned} d_{tu(P)} &= \mathbb{E}_{(P)}\left(\eta_u\left(\boldsymbol{X},\boldsymbol{U}\right) - \eta_t\left(\boldsymbol{X},\boldsymbol{U}\right)\right) \\ &= \beta_u - \beta_t + \mathbb{E}_{(P)}\left(\boldsymbol{\beta}_2^T\boldsymbol{X}^{EM} + \boldsymbol{\varphi}_2^T\boldsymbol{U}^{EM}\right) - \mathbb{E}_{(P)}\left(\boldsymbol{\beta}_2^T\boldsymbol{X}^{EM} + \boldsymbol{\varphi}_2^T\boldsymbol{U}^{EM}\right) \\ &= \beta_u - \beta_t \end{aligned}$$

$$\begin{aligned} d_{tu(Q)} &= \mathbb{E}_{(Q)}\left(\eta_u\left(\boldsymbol{X},\boldsymbol{U}\right) - \eta_t\left(\boldsymbol{X},\boldsymbol{U}\right)\right) \\ &= \beta_u - \beta_t + \mathbb{E}_{(Q)}\left(\boldsymbol{\beta}_2^T\boldsymbol{X}^{EM} + \boldsymbol{\varphi}_2^T\boldsymbol{U}^{EM}\right) - \mathbb{E}_{(Q)}\left(\boldsymbol{\beta}_2^T\boldsymbol{X}^{EM} + \boldsymbol{\varphi}_2^T\boldsymbol{U}^{EM}\right) \\ &= \beta_u - \beta_t \end{aligned}$$

Therefore $d_{tu(P)} = d_{tu(Q)}$ holds for all $t,u \in \mathcal{T}$.

EXAMPLE

To see the application of the shared effect modifier assumption in practice, consider an example where the following log odds ratios in the AC population have been estimated to be $\hat{d}_{AB(AC)} = 1.3$ and $\hat{d}_{AC(AC)} = 0.8$, and therefore $\hat{d}_{BC(AC)} = \hat{d}_{AC(AC)} - \hat{d}_{AB(AC)} = -0.5$.

Furthermore, in a population P the log odds ratio for treatment B compared to A is estimated to be $\hat{d}_{AB(P)} = 0.7$. We make the shared effect modifier assumption for treatments $\{B, C\} = \mathcal{T}$.

With the shared effect modifier assumption, we use relation (9) to see that $\hat{d}_{BC(P)} = \hat{d}_{BC(AC)} = -0.5$, and the log odds ratio for treatment C compared to A in population P is inferred to be $\hat{d}_{AC(P)} = \hat{d}_{AB(P)} + \hat{d}_{BC(P)} = 0.2$.

Appendix B: REPORTING OF POPULATION-ADJUSTED ANALYSES

When reporting population-adjusted analyses, the following themes should be considered and addressed explicitly:

1. The variables available in each study should be listed, along with their distributions (e.g. through box plots or histograms). Sufficient covariate overlap between the populations should be assessed: for population reweighting methods (such as MAIC), the number of individuals assigned zero weight should be reported; for outcome regression methods (such as STC), the amount of extrapolation required should be considered. For anchored comparisons this applies only to effect modifiers (see point 2); for unanchored comparisons all variables relevant to outcome should be presented.
2. Evidence for effect modifier status should be given, along with the proposed size of the interaction effect and the imbalance between the study populations. The resulting potential bias reduction compared with a standard indirect comparison may be calculated by multiplying the interaction coefficient by the difference in means.[10]
3. The distribution of weights should be presented for population weighting analyses, and used to highlight any issues with extreme or highly variable weights. Presentation of the effective sample size may also be useful. ESS may be approximated using equation (7) – which is likely to be an underestimate – but provides clear warning where inferences are being made based on just a small number of individuals.
4. Measures of uncertainty, such as confidence intervals, should always be presented alongside any estimates. Care should be taken that uncertainty is appropriately propagated through to the final estimates. For outcome regression methods, uncertainty is fully propagated for predictions into the aggregate population by the outcome regression model. For population reweighting methods, a robust sandwich estimator (as typical for MAIC) provides estimates of standard error which account for all sources of uncertainty. Other techniques include bootstrapping and Bayesian methods.
5. For an unanchored comparison, estimates of systematic error before and after population adjustment should be presented.[10]
6. Present estimates for the appropriate target population using the shared effect modifier assumption if appropriate, or comment on the representativeness of the aggregate population to the true target population.
7. In order to convey some clarity about the impact of any population adjustment, the standard indirect comparison estimate should be presented alongside the population-adjusted indirect

comparison if an anchored comparison is formed; for an unanchored comparison, a crude unadjusted difference should be presented alongside the MAIC/STC estimate.

Appendix C: PROCESS FOR POPULATION-ADJUSTED INDIRECT COMPARISONS

Figure 1: Anchored methods for population-adjusted indirect comparisons

Propensity score reweighting | **Outcome regression**

1. Provide evidence for effect modifier status on a suitable transformed scale.

2. Provide evidence that effect modifiers are in substantial imbalance between studies.

Propensity score reweighting:

3a. Create a logistic propensity score model, which includes all effect modifiers but no prognostic variables. This is equivalent to a model on the log of the weights:

$$\log\left(w_{it}\right) = \alpha_0 + \boldsymbol{\alpha}_1^T \boldsymbol{X}_{it}^{EM}$$

3b. Estimate the weights using the method of moments to match effect modifier distributions between trials. This is equivalent to minimising

$$\sum_{t=A,B} \sum_{i=1}^{N_{t(AB)}} \exp\left(\boldsymbol{\alpha}_1^T \boldsymbol{X}_{it}^{EM}\right)$$

when $\bar{\boldsymbol{X}}_{(AC)}^{EM} = \boldsymbol{0}$.

Outcome regression:

3. Fit an outcome model in the AB trial, which includes all effect modifiers in imbalance and any other prognostic variables or effect modifiers that improve model fit:

$$g\left(\mu_{t(AB)}\left(\boldsymbol{X}\right)\right) = \beta_0 + \boldsymbol{\beta}_1^T \boldsymbol{X} + \left(\beta_B + \boldsymbol{\beta}_2^T \boldsymbol{X}^{EM}\right) I\left(t = B\right)$$

Propensity score reweighting:

4. Predict outcomes on treatments A and B in the AC trial by reweighting the outcomes of the AB individuals:

$$\hat{Y}_{t(AC)} = \frac{\sum_{i=1}^{N_{t(AB)}} Y_{it(AB)} \hat{w}_{it}}{\sum_{i=1}^{N_{t(AB)}} \hat{w}_{it}}$$

Outcome regression:

4. Predict transformed outcomes on treatments A and B in the AC trial using the outcome model:

$$g\left(\hat{Y}_{t(AC)}\right) = \hat{\beta}_0 + \hat{\boldsymbol{\beta}}_1^T \bar{\boldsymbol{X}}_{(AC)} + \left(\hat{\beta}_B + \hat{\boldsymbol{\beta}}_2^T \bar{\boldsymbol{X}}_{(AC)}^{EM}\right) I\left(t = B\right)$$

5. Form the anchored indirect comparison in the AC population as:

$$\hat{\Delta}_{BC(AC)} = g\left(\bar{Y}_{C(AC)}\right) - g\left(\bar{Y}_{A(AC)}\right) - \left(g\left(\hat{Y}_{B(AC)}\right) - g\left(\hat{Y}_{A(AC)}\right)\right)$$

Propensity score reweighting:

6. Calculate standard errors using a robust sandwich estimator, bootstrapping, or Bayesian techniques.

Outcome regression:

6. Calculate standard errors using the outcome model.

7. If justified, use the shared effect modifier assumption to transport the $\hat{\Delta}_{BC(AC)}$ estimate into the target population for the decision. Otherwise, comment on the representativeness of the AC population to the true target population.

Propensity score reweighting:

8. Present the distribution of estimated weights, and effective sample size.

Outcome regression:

8. Present standard model fit statistics.

Figure 2: Unanchored methods for population-adjusted indirect comparisons

Propensity score reweighting

1a. Create a logistic propensity score model, which includes all effect modifiers and prognostic variables. This is equivalent to a model on the log of the weights:

$$\log\left(w_i\right) = \alpha_0 + \boldsymbol{\alpha}_1^T \boldsymbol{X}_i$$

1b. Estimate the weights using the method of moments to match effect modifier distributions between trials. This is equivalent to minimising

$$\sum_{i=1}^{N_{B(B)}} \exp\left(\boldsymbol{\alpha}_1^T \boldsymbol{X}_i\right)$$

when $\overline{\boldsymbol{X}}_{(C)}^{EM} = \boldsymbol{0}$.

2. Predict outcomes on treatment B in the C trial by reweighting the outcomes of the B individuals:

$$\hat{Y}_{B(C)} = \frac{\sum_{i=1}^{N_{B(B)}} Y_{i(B)} \hat{w}_i}{\sum_{i=1}^{N_{B(B)}} \hat{w}_i}$$

Outcome regression

1. Fit an outcome model in the B trial, which includes all effect modifiers and prognostic variables:

$$g\left(\mu_{B(B)}\left(\boldsymbol{X}\right)\right) = \beta_0 + \boldsymbol{\beta}_1^T \boldsymbol{X} + \left(\beta_B + \boldsymbol{\beta}_2^T \boldsymbol{X}^{EM}\right)$$

2. Predict transformed outcomes on treatments A and B in the C trial using the outcome model:

$$g\left(\hat{Y}_{B(C)}\right) = \hat{\beta}_0 + \hat{\boldsymbol{\beta}}_1^T \overline{\boldsymbol{X}}_{(C)} + \left(\hat{\beta}_B + \hat{\boldsymbol{\beta}}_2^T \overline{\boldsymbol{X}}_{(C)}^{EM}\right)$$

3. Form the unanchored indirect comparison in the C population as:

$$\hat{\Delta}_{BC(C)} = g\left(\overline{Y}_{C(C)}\right) - g\left(\hat{Y}_{B(C)}\right)$$

Propensity score reweighting

4. Calculate standard errors using a robust sandwich estimator, bootstrapping, or Bayesian techniques.

Outcome regression

4. Calculate standard errors using the outcome model.

5. Provide evidence that absolute outcomes can be predicted with sufficient accuracy in relation to the relative treatment effects, and present an estimate of the likely range of residual systematic error. If this evidence cannot be provided or is limited, then state that the amount of bias in the indirect comparison is likely to be substantial, and could even exceed the magnitude of treatment effects which are being estimated.

6. If justified, use the shared effect modifier assumption to transport the $\hat{\Delta}_{BC(C)}$ estimate into the target population for the decision. Otherwise, comment on the representativeness of the C population to the true target population.

Propensity score reweighting

7. Present the distribution of estimated weights, and effective sample size.

Outcome regression

7. Present standard model fit statistics.
