# Supplementary material for: Methods for Population-Adjusted Indirect Comparisons in Health Technology Appraisal
Source: Med Decis Making. 2017 Aug 19;38(2):200–11. doi: 10.1177/0272989X17725740 (PMC5774635; doi:10.1177/0272989X17725740)
Supplement: Supplementary material [file Example.pdf]

Worked example of MAIC and STC

We provide worked examples of anchored forms of MAIC and STC in a simple two-study scenario (AB IPD study, AC aggregate study). For simplicity we include only two covariates, age and gender, and age will act as an effect modifier.

We load the following packages, although STC can be carried out entirely in base R, and MAIC requires only the `sandwich` package (to easily obtain standard errors using a sandwich estimator). The other packages are loaded to simplify data simulation and manipulation, and to provide graphics.

```
if(!require(dplyr)) {install.packages("dplyr"); library(dplyr)}
## Loading required package: dplyr
##
## Attaching package: 'dplyr'
## The following objects are masked from 'package:stats':
##
##     filter, lag
## The following objects are masked from 'package:base':
##
##     intersect, setdiff, setequal, union
if(!require(tidyr)) {install.packages("tidyr"); library(tidyr)}
## Loading required package: tidyr
if(!require(wakefield)) {install.packages("wakefield"); library(wakefield)}
## Loading required package: wakefield
##
## Attaching package: 'wakefield'
## The following object is masked from 'package:dplyr':
##
##     id
if(!require(ggplot2)) {install.packages("ggplot2"); library(ggplot2)}
## Loading required package: ggplot2
if(!require(sandwich)) {install.packages("sandwich"); library(sandwich)}
## Loading required package: sandwich
```

Creating simulated datasets

First, we create some simulated data. We use the package `wakefield`, which provides an easy way to quickly create realistic simulated datasets with pre-set variable types. We will consider two variables, age and gender, with age being an effect modifier and gender a purely prognostic variable.

Here, we set the parameters of the simulation scenario, defining study characteristics (study sizes, age ranges, proportion of females) and the parameters of the outcome model. We also set a seed value for reproducibility.

```
set.seed(61374988)

# Study characteristics
N_AB <- 500
N_AC <- 300
agerange_AB <- 45:75
agerange_AC <- 45:55
femalepc_AB <- 0.64
femalepc_AC <- 0.8
```

```r
# Outcome model
b_0 <- 0.85
b_gender <- 0.12
b_age <- 0.05
b_age_trt <- -0.08
b_trt_B <- -2.1
b_trt_C <- -2.5
```

For our example, we will generate binary outcomes. The true logistic outcome model which we use to simulate the data will be:

$$\text{logit}(p_{it}) = 0.85 + 0.12 \cdot male_{it} + 0.05 \cdot (age_{it} - 40) + (\beta_t + -0.08 \cdot (age_{it} - 40))\mathbb{I}(t \neq A)$$

where $\beta_B = -2.1$ and $\beta_C = -2.5$. The parameters of the model are interpreted as log odds ratios, and p_{it} is the probability of an event for individual i receiving treatment t.

AB trial

The AB trial ($N_{(AB)} = 500$) will have ages from 45 to 75 and 64% females. The `wakefield` package provides a framework for generating simulated datasets, including many convenience functions; we make use of just a small number here, including `age` to uniformly generate ages within an age range, `gender` to generate a factor variable of genders with given probabilities, and `id` to create a unique ID for each individual.

```r
AB.IPD <-
  rbind(

  # Generate A arm
  r_data_frame(n = N_AB/2,     # Number of individuals in arm A
               id,             # Unique ID
               age = age(x = agerange_AB),   # Generate ages
               gender = gender(prob = c(1 - femalepc_AB, femalepc_AB)), # Generate genders
               trt = "A"       # Assign treatment A
               ),

  # Generate B arm
  r_data_frame(n = N_AB/2,     # Number of individuals in arm B
               id,             # Unique ID
               age = age(x = agerange_AB),   # Generate ages
               gender = gender(prob = c(1 - femalepc_AB, femalepc_AB)), # Generate genders
               trt = "B"       # Assign treatment B
               )
  ) %>%

  # Generate outcomes using logistic model
  mutate(
    yprob = 1 / (1 + exp(-(
      b_0 + b_gender * (gender == "Male") + b_age * (age - 40) +
        if_else(trt == "B", b_trt_B + b_age_trt * (age - 40), 0)
    ))),
    y = rbinom(N_AB, 1, yprob)
  ) %>%
  select(-yprob)   # Drop the yprob column
```

Tabulate the AB trial, to check that our “randomisation” has worked, and examine the generated outcomes.

```
AB.IPD %>% group_by(trt) %>%
  summarise(n(), mean(age), sd(age), `n(male)`=sum(gender=="Male"),
            `%(male)`=mean(gender=="Male"), sum(y), mean(y))
```

```
## # A tibble: 2 × 8
##     trt `n()` `mean(age)` `sd(age)` `n(male)` `%(male)` `sum(y)` `mean(y)`
##   <chr> <int>       <dbl>     <dbl>     <int>     <dbl>    <int>     <dbl>
## 1     A   250      60.016  9.152421        87     0.348      217     0.868
## 2     B   250      60.084  9.208699        92     0.368       39     0.156
```

AC trial

The *AC* trial ($N_{(AC)} = 300$) will have ages from 45 to 55 and 80% females.

```
AC.IPD <-
  rbind(

  # Generate A arm
  r_data_frame(n = N_AC/2,      # Number of individuals in arm A
               id,              # Unique ID
               age = age(x = agerange_AC),    # Generate ages
               gender = gender(prob = c(1 - femalepc_AC, femalepc_AC)), # Generate genders
               trt = "A"        # Assign treatment A
               ),

  # Generate C arm
  r_data_frame(n = N_AC/2,      # Number of individuals in arm C
               id,              # Unique ID
               age = age(x = agerange_AC),    # Generate ages
               gender = gender(prob = c(1 - femalepc_AC, femalepc_AC)), # Generate genders
               trt = "C"        # Assign treatment C
               )
  ) %>%

  # Generate outcomes using logistic model
  mutate(
    yprob = 1 / (1 + exp(-(
      b_0 + b_gender * (gender == "Male") + b_age * (age - 40) +
        if_else(trt == "C", b_trt_C + b_age_trt * (age - 40), 0)
    ))),
    y = rbinom(N_AC, 1, yprob)
  ) %>%
  select(-yprob)    # Drop the yprob column
```

Tabulate the *AC* trial, to check that our “randomisation” has worked, and examine the generated outcomes.

```
AC.IPD %>% group_by(trt) %>%
  summarise(n(), mean(age), sd(age), `n(male)`=sum(gender=="Male"),
            `%(male)`=mean(gender=="Male"), sum(y), mean(y))
```

```
## # A tibble: 2 × 8
##     trt `n()` `mean(age)` `sd(age)` `n(male)` `%(male)` `sum(y)` `mean(y)`
##   <chr> <int>       <dbl>     <dbl>     <int>     <dbl>    <int>     <dbl>
## 1     A   150    49.50667  2.955479        33      0.22      115 0.7666667
## 2     C   150    50.10667  3.185847        27      0.18       17 0.1133333
```

For analysis we will have access only to the aggregate data, as if from a published study. Here, we aggregate the IPD to obtain summaries, which will be used for the MAIC and STC analyses.

```
AC.AgD <-
  cbind(
    # Trial level stats: mean and sd of age, number and proportion of males
    summarise(AC.IPD, age.mean = mean(age), age.sd = sd(age),
              N.male = sum(gender=="Male"), prop.male = mean(gender=="Male")),

    # Summary outcomes for A arm
    filter(AC.IPD, trt == "A") %>%
      summarise(y.A.sum = sum(y), y.A.bar = mean(y), N.A = n()),

    # Summary outcomes for C arm
    filter(AC.IPD, trt == "C") %>%
      summarise(y.C.sum = sum(y), y.C.bar = mean(y), N.C = n())
  )

AC.AgD
```

```
##   age.mean   age.sd N.male prop.male y.A.sum   y.A.bar N.A y.C.sum
## 1 49.80667 3.082363     60       0.2     115 0.7666667 150      17
##     y.C.bar N.C
## 1 0.1133333 150
```

MAIC

We are now ready to proceed with our analyses. First, we will estimate the population-adjusted indirect comparison using MAIC. This involves estimating a logistic propensity score model, which includes all effect modifiers but no prognostic variables. This is equivalent to the following model on the log of the individual weights:

$$\log(w_{it}) = \alpha_0 + \boldsymbol{\alpha}_1^T \mathbf{X}_{it}^{EM}$$

The weights are estimated using the method of moments to match the effect modifier distributions between the AB and AC trials. This is equivalent to minimising

$$\sum_{i,t} \exp(\boldsymbol{\alpha}_1^T \mathbf{X}_{it}^{EM})$$

when $\bar{\mathbf{X}}_{(AC)}^{EM} = 0$.

In order to do this, we define the objective function to minimise (as above), and the gradient function (its derivative) which will be used by the minimisation algorithm.

```
objfn <- function(a1, X){
  sum(exp(X %*% a1))
}

gradfn <- function(a1, X){
  colSums(sweep(X, 1, exp(X %*% a1), "*"))
}
```

To satisfy $\bar{\mathbf{X}}_{(AC)}^{EM} = 0$, we create centred versions of the effect modifiers by subtracting $\bar{\mathbf{X}}_{(AC)}^{EM}$ from $\mathbf{X}^{EM}$ in both trials. Here only age is an effect modifier, and we can balance this in both mean and standard deviation as we have observed `age.mean` and `age.sd` in the AC trial. We therefore include centred versions of `age` and `age^2` for each individual in the AB trial in the weighting model. Centring the mean is simple,

but centring higher moments requires some attention: due to aggregation, we cannot simply centre `age^2` from the AB trial with `age.sd` from the AC trial. We use the variance formula $\text{var}(\mathbf{X}) = \mathbb{E}(\mathbf{X}^2) - \mathbb{E}(\mathbf{X})^2$, and centre `age^2` in the AB trial with `age.mean^2 + age.sd^2` in the AC trial. Here we make use of the `sweep` function to simultaneously centre the two row vectors `age` and `age^2` by subtracting `age.mean` and `age.mean^2 + age.sd^2` respectively:

```
X.EM.0 <- sweep(with(AB.IPD, cbind(age, age^2)), 2,
                with(AC.AgD, c(age.mean, age.mean^2 + age.sd^2)), '-')
```

To estimate $\boldsymbol{\alpha}_1$, we use the function `optim` to minimise the function `objfn`. The method we tell `optim` to use is BFGS (after Broyden, Fletcher, Goldfarb and Shanno), which makes use of the gradient function `gradfn` that we specified to aid minimisation. We have to specify an initial value in the `par` argument (we choose `c(0,0)` here), and `X = X.EM.0` is passed to `objfn` and `gradfn` as an additional argument.

```
print(opt1 <- optim(par = c(0,0), fn = objfn, gr = gradfn, X = X.EM.0, method = "BFGS"))
```

```
## $par
## [1]  3.15553663 -0.03224845
##
## $value
## [1] 202.1651
##
## $counts
## function gradient
##       67       14
##
## $convergence
## [1] 0
##
## $message
## NULL
```

```
a1 <- opt1$par
```

The output generated simply states that convergence has occurred successfully (`$convergence` $= 0$). The estimate $\hat{\boldsymbol{\alpha}}_1$ is found in `$par`. (The other outputs are `$value`, the value of `objfn` at the minimum, `$counts`, the number of evaluations of `objfn` and `gradfn` before convergence, and `$message`, for any additional information from the minimisation algorithm.)

The estimated weights for each individual are then found by $\hat{w_{it}} = \exp\left(\bar{\mathbf{X}}_{it}^{EM}\hat{\alpha}_1\right)$. We do not need to estimate α_0, as this constant cancels out.

```
wt <- exp(X.EM.0 %*% a1)
```

It is easier to examine the distribution of the weights by scaling them, so that the rescaled weights are relative to the original unit weights of each individual; in other words, a rescaled weight > 1 means that an individual carries more weight in the reweighted population than in the AB population, and a rescaled weight < 1 means that an individual carries less weight. The rescaled weight is calculated as

$$\tilde{w}_{it} = \frac{\hat{w}_{it}}{\sum_{i,t}\hat{w}_{it}} \cdot N_{(AB)}$$

```
wt.rs <- (wt / sum(wt)) * N_AB   # rescaled weights

summary(wt.rs)
```

```
##          V1
##  Min.   :0.000000
```

```
##   1st Qu.:0.000023
##   Median :0.065993
##   Mean   :1.000000
##   3rd Qu.:2.096045
##   Max.   :3.444506
```

```
qplot(wt.rs, geom="histogram",
      xlab = "Rescaled weight (multiple of original unit weight)",
      binwidth=0.25)
```

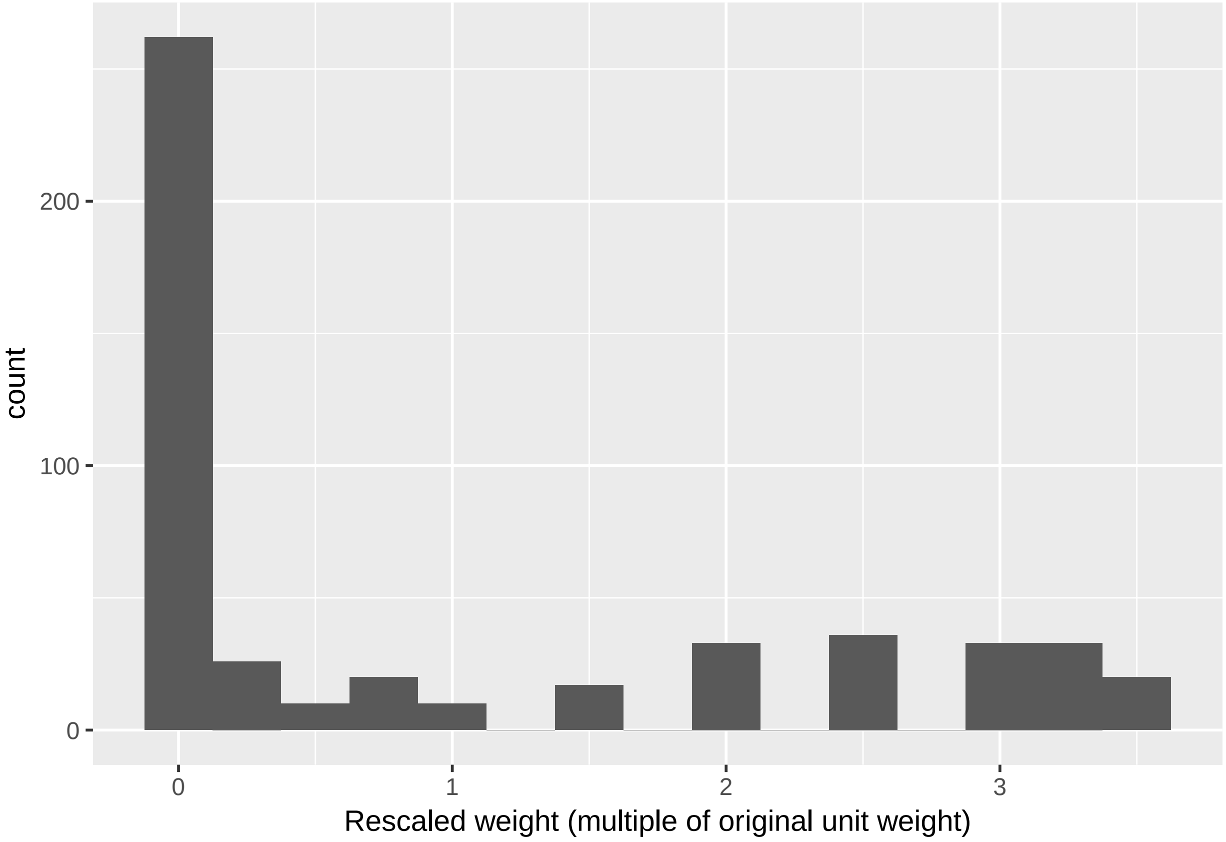

The mean of the rescaled weights is not informative, as it is guaranteed to be 1:

$$\frac{1}{N_{(AB)}} \sum_{i,t} \tilde{w}_{it} = \frac{1}{N_{(AB)}} \sum_{i,t} \frac{\hat{w}_{it}}{\sum_{i,t} \hat{w}_{it}} \cdot N_{(AB)} = 1$$

Here, the rescaled weights range from 0 to 3.44, and the median is heavily skewed towards zero (0.07). A histogram of the weights is also very helpful to present, and clearly shows that a large number of individuals have been given zero (or close to zero) weight. This is not surprising, as the age range in the AB trial (45 to 75) is much wider than that in the AC trial (45 to 55). There are therefore a large number of individuals from the AB trial who have been excluded. More positively, there are no very large weights, as the distribution of effect modifiers in the AC population is entirely contained within that of the AB population (there are no ages in the AC population outside of those observed in the AB population).

The approximate effective sample size is calculated as

$$\frac{\sum_{i,t} (\hat{w}_{it})^2}{\sum_{i,t} \hat{w}_{i,t}^2}$$

```r
sum(wt)^2/sum(wt^2)
```

```
## [1] 185.6451
```

This is quite a reduction from the original 500, but is still reasonably large. (The actual ESS is likely to be larger than this, as the weights are not fixed and known.)

Note that age is balanced (in terms of mean and SD) with the AC population after weighting.

```r
AB.IPD %>%
  mutate(wt) %>%
  summarise(age.mean = weighted.mean(age, wt),
            age.sd = sqrt(sum(wt / sum(wt) * (age - age.mean)^2))
            )
```

```
## # A tibble: 1 × 2
##   age.mean   age.sd
##      <dbl>    <dbl>
## 1 49.80667 3.082363
```

```r
AC.AgD[, c("age.mean", "age.sd")]
```

```
##   age.mean   age.sd
## 1 49.80667 3.082363
```

The estimated relative effect $\hat{d}_{AB(AC)}$ of B vs. A in the AC population is found by taking weighted means of the outcomes in the AB trial. However, in practice it is easier to generate these estimates using a simple linear model: this is exactly equivalent to taking the weighted means, but allows us to use the `sandwich` package to calculate standard errors correctly using a sandwich estimator. (Note that it is possible to generate estimates of absolute outcomes on each treatment using the weighted means or the linear model, but these *will be biased* unless all prognostic variables and effect modifiers in imbalance between the populations are accounted for. Unbiased prediction of absolute outcomes relies on the much stronger assumption of conditional constancy of absolute effects.)

```r
# Binomial GLM
fit1 <-
  AB.IPD %>% mutate(y0 = 1 - y, wt = wt) %>%
  glm(cbind(y,y0) ~ trt, data = ., family = binomial, weights = wt)

# Sandwich estimator of variance matrix
V.sw <- vcovHC(fit1)

# The log OR of B vs. A is just the trtB parameter estimate,
# since effect modifiers were centred
print(d.AB.MAIC <- coef(fit1)["trtB"])
```

```
##      trtB
## -3.215136
```

```r
print(var.d.AB.MAIC <- V.sw["trtB","trtB"])
```

```
## [1] 0.1628077
```

Finally, we construct the indirect comparison estimate $\hat{d}_{BC(AC)}$ on the log OR scale, using the consistency relation $d_{BC(AC)} = d_{AC(AC)} - d_{AB(AC)}$.

```r
# Estimated log OR of C vs. A from the AC trial
d.AC <- with(AC.AgD, log(y.C.sum * (N.A - y.A.sum) / (y.A.sum * (N.C - y.C.sum))))
var.d.AC <- with(AC.AgD, 1/y.A.sum + 1/(N.A - y.A.sum) + 1/y.C.sum + 1/(N.C - y.C.sum))
```

```r
# Indirect comparison
print(d.BC.MAIC <- d.AC - d.AB.MAIC)
```

```
##        trtB
## -0.03158391
```

```r
print(var.d.BC.MAIC <- var.d.AC + var.d.AB.MAIC)
```

```
## [1] 0.2664171
```

So the MAIC estimate of the log odds ratio of treatment C vs. B is -0.032, with standard error $\sqrt{0.266} = 0.516$. We examine this result in more detail later on in the summary section.

STC

Alternatively, we can use STC to make the indirect comparison, which involves creating an outcome regression model. We fit a regression model in the AB trial population, and use this to predict outcomes in the AC trial population. The outcome model need only contain all effect modifiers in imbalance in order to be unbiased, but adding other covariates may increase precision. In this case, we only need to include age in the model, but adding gender may increase precision by accounting for more of the variation.

First, fit the outcome model. If we centre `age` at the mean value from the AC population, then the interpretation of the `trtB` coefficient is the average B vs. A effect in the AC population.

```r
AB.IPD$y0 <- 1 - AB.IPD$y  # Add in dummy non-event column

# Fit binomial GLM
STC.GLM <- glm(cbind(y,y0) ~ trt*I(age - AC.AgD$age.mean),
               data = AB.IPD, family = binomial)
summary(STC.GLM)
```

```
##
## Call:
## glm(formula = cbind(y, y0) ~ trt * I(age - AC.AgD$age.mean),
##     family = binomial, data = AB.IPD)
##
## Deviance Residuals:
##     Min       1Q   Median       3Q      Max
## -2.2617  -0.5932   0.4016   0.5426   2.0739
##
## Coefficients:
##                               Estimate Std. Error z value Pr(>|z|)
## (Intercept)                    1.54856    0.25205   6.144 8.05e-10 ***
## trtB                          -2.99962    0.35089  -8.549  < 2e-16 ***
## I(age - AC.AgD$age.mean)       0.03685    0.02103   1.752   0.0797 .
## trtB:I(age - AC.AgD$age.mean) -0.06167    0.02851  -2.163   0.0305 *
## ---
## Signif. codes:  0 '***' 0.001 '**' 0.01 '*' 0.05 '.' 0.1 ' ' 1
##
## (Dispersion parameter for binomial family taken to be 1)
##
##     Null deviance: 692.86  on 499  degrees of freedom
## Residual deviance: 406.73  on 496  degrees of freedom
## AIC: 414.73
##
```

```
## Number of Fisher Scoring iterations: 4
```

```r
# Try adding prognostic variables to improve model fit
add1(STC.GLM, ~.+gender, test="Chisq")
```

```
## Single term additions
##
## Model:
## cbind(y, y0) ~ trt * I(age - AC.AgD$age.mean)
##        Df Deviance    AIC     LRT Pr(>Chi)
## <none>      406.73 414.73
## gender  1   406.33 416.33 0.40192   0.5261
```

The residual deviance suggests that the model fits the data well, as `pchisq`(406.73, 496) = 0.001. However, even if the model fit was poor, the eventual indirect comparison would still be unbiased – as long as all effect modifiers in imbalance are included in the model, so that conditional constancy of relative effects is satisfied. It is most important that effect modifiers are pre-specified prior to analysis (as per the NICE Methods Guide, and ISPOR guidance); if this is done properly, and all effect modifiers are identified, then the reliance on traditional "model checking" is greatly reduced. Variable selection techniques should not be used to justify the inclusion or exclusion of effect modifiers in the model.

We may however try to add other prognostic variables to the model, to try and improve model fit and possibly reduce the standard error of the indirect comparison. Adding gender to the model did not alter the residual deviance or the AIC significantly, so we continue without gender in the model.

(Note that not all of the estimated coefficients match those of the true underlying logistic model which we specified earlier. This is because some of their interpretations are different: the reference age here is the mean age of the AC population, whereas the reference age for the true model was age 40.)

The log OR $d_{AB(AC)}$ of B vs. A in the AC population is then estimated from the model:

```r
print(d.AB.STC <- coef(STC.GLM)["trtB"])
```

```
##     trtB
## -2.99962
```

```r
print(var.d.AB.STC <- vcov(STC.GLM)["trtB","trtB"])
```

```
## [1] 0.1231246
```

Finally, we construct the indirect comparison $d_{BC(AC)}$ on the log OR scale:

```r
print(d.BC.STC <- d.AC - d.AB.STC)
```

```
##       trtB
## -0.2470995
```

```r
print(var.d.BC.STC <- var.d.AC + var.d.AB.STC)
```

```
## [1] 0.226734
```

So the STC estimate of the log odds ratio of treatment C vs. B is -0.247, with standard error $\sqrt{0.227} = 0.476$. We examine this result and that from MAIC in more detail below.

Summary

The true B vs. A effect (log OR) in the AC population is calculated as

```r
d.AB.TRUE <- b_trt_B + b_age_trt * (AC.AgD$age.mean - 40)
```

The naive approach would be to just transport the estimated B vs. A effect from the AB population directly, ignoring any effect modifiers in imbalance:

```
AB.IPD %>% group_by(trt) %>%
summarise(y.sum = sum(y)) %>%
spread(trt, y.sum) %>%
  with({
    d.AB.AB <<- log(B * (N_AB/2 - A) / (A * (N_AB/2 - B)))
    var.d.AB.AB <<- 1/B + 1/(N_AB/2 - A) + 1/A + 1/(N_AB/2 - B)
  })
```

Tabulating the various B vs. A estimates in the AC population:

IC of B vs. A	Estimate	95% CI
d.AB.TRUE	-2.885	-
d.AB.MAIC	-3.215	(-4.006, -2.424)
d.AB.STC	-3.000	(-3.687, -2.312)
d.AB.AB	-3.572	(-4.073, -3.071)

We see that both MAIC and STC have provided estimates of the B vs. A effect in the AC population that have reduced bias compared to the naively transported estimate from the AB population. The confidence intervals of both the MAIC and STC estimates both include the true value, whereas the confidence interval for the naive estimate does not.

The true C vs. B effect in the AC population is calculated as

```
d.BC.TRUE <- b_trt_C - b_trt_B
```

A naive indirect comparison, ignoring the presence of effect modifiers, would calculate the C vs. B effect in the AC population as

```
d.BC.NAIVE <- d.AC - d.AB.AB
var.d.BC.NAIVE <- var.d.AC + var.d.AB.AB
```

Tabulating the various C vs. B estimates in the AC population:

IC of C vs. B	Estimate	95% CI
d.BC.TRUE	-0.400	-
d.BC.MAIC	-0.032	(-1.043, 0.980)
d.BC.STC	-0.247	(-1.180, 0.686)
d.BC.NAIVE	0.325	(-0.481, 1.130)

The estimates from MAIC and STC are much closer to the true value than the naive unadjusted indirect comparison, and their confidence intervals include the true value. We can visualise the various estimates in the AC population on a forest plot:

```
plotdat <- data_frame(
  id = 1:10,
  Comparison = factor(c(rep(1,4), 2, 2, rep(3,4)),
                      labels = c("B vs. A", "C vs. A", "C vs. B")),
  Estimate = c(d.AB.TRUE, d.AB.MAIC, d.AB.STC, d.AB.AB,
               b_trt_C + b_age_trt * (AC.AgD$age.mean - 40), d.AC,
               d.BC.TRUE, d.BC.MAIC, d.BC.STC, d.BC.NAIVE),
  var = c(NA, var.d.AB.MAIC, var.d.AB.STC, var.d.AB.AB,
          NA, var.d.AC,
```

```
          NA, var.d.BC.MAIC, var.d.BC.STC, var.d.BC.NAIVE),
  lo = Estimate + qnorm(0.025) * sqrt(var),
  hi = Estimate + qnorm(0.975) * sqrt(var),
  type = c("True", "MAIC", "STC", "Unadjusted",
           "True","Unadjusted",
           "True", "MAIC", "STC", "Unadjusted")
)

ggplot(aes(x = Estimate, y = id, col = type, shape = type), data = plotdat) +
  geom_vline(xintercept = 0, lty = 2) +
  geom_point(size = 2) +
  geom_segment(aes(y = id, yend = id, x = lo, xend = hi), na.rm = TRUE) +
  xlab("Estimate (Log OR)") +
  facet_grid(Comparison~., switch = "y", scales = "free_y", space = "free_y") +
  scale_y_reverse(name = "Comparison in AC population", breaks = NULL, expand = c(0, 0.6))
```

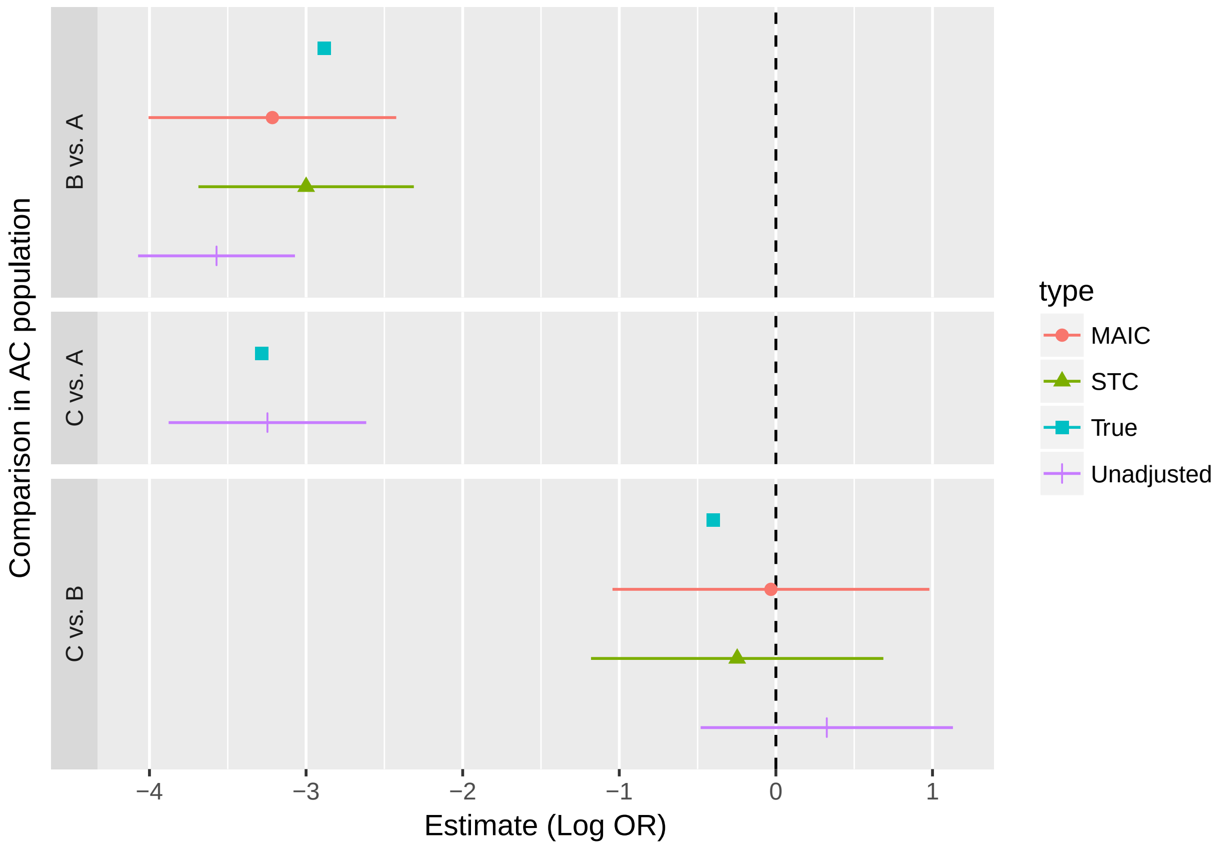

Here we can clearly see how MAIC and STC have successfully reduced the bias in an anchored comparison compared to the standard "unadjusted" approach. Naively transporting the B vs. A estimate from the AB trial results in large bias; the ensuing C vs. B indirect comparison even has the wrong sign, and incorrectly favours treatment B over treatment C in the AC population. The MAIC and STC estimates lie the correct side of zero, and their 95% confidence intervals include the true value.

Whilst in this scenario STC appears to have produced an estimate closer to the true value and with a slightly narrower confidence interval, these results should *not* be taken in any way to mean that either method is preferred. The properties and performance of MAIC and STC (and other methods) need to be thoroughly investigated through simulation studies, particularly under failure of the requisite assumptions. Then, and

only then, can preferences be expressed for any given method.
